# Fertility preservation and in vitro fertilization (IVF) success rates after cancer

**DOI:** 10.1093/jncics/pkaf057

**Published:** 2025-06-10

**Authors:** Chelsea Anderson, Alexis C Wardell, Allison M Deal, Jennifer E Mersereau, Katie Cameron, Steven D Spandorfer, Valerie L Baker, Sara Mitra, Jianwen Cai, Barbara Luke, Hazel B Nichols

**Affiliations:** Center for Gastrointestinal Biology and Disease, University of North Carolina at Chapel Hill School of Medicine, Chapel Hill, NC, United States; Lineberger Comprehensive Cancer Center, School of Medicine, University of North Carolina at Chapel Hill, Chapel Hill, NC, United States; Lineberger Comprehensive Cancer Center, School of Medicine, University of North Carolina at Chapel Hill, Chapel Hill, NC, United States; Shady Grove Fertility, Carolinas, Cary, NC, United States; Division of Reproductive Endocrinology and Infertility, Department of Gynecology and Obstetrics, Johns Hopkins University School of Medicine, Baltimore, MD, United States; The Center for Reproductive Medicine and Infertility, Cornell University Medical Center, New York, NY, United States; Division of Reproductive Endocrinology and Infertility, Department of Gynecology and Obstetrics, Johns Hopkins University School of Medicine, Baltimore, MD, United States; Lineberger Comprehensive Cancer Center, School of Medicine, University of North Carolina at Chapel Hill, Chapel Hill, NC, United States; Department of Biostatistics, University of North Carolina at Chapel Hill Gillings School of Global Public Health, Chapel Hill, NC, United States; Department of Obstetrics, Gynecology, and Reproductive Biology, College of Human Medicine, Michigan State University, East Lansing, MI, United States; Department of Epidemiology, University of North Carolina Gillings School of Global Public Health, Chapel Hill, NC, United States

## Abstract

**Background:**

Evidence of the success of in vitro fertilization (IVF) procedures is critical for informed decision making before and after cancer treatment. We compared IVF outcomes between women with and without cancer.

**Methods:**

Using data from a national IVF database—the Society for Assisted Reproductive Clinic Outcomes Reporting System, linked to statewide cancer registries and birth certificates in 9 states—we identified women who initiated IVF after a cancer diagnosis. Fertility preservation was defined as oocyte retrieval ≤90 days after cancer diagnosis, and IVF after cancer treatment as retrieval >90 days postdiagnosis. Number of oocytes retrieved and conception and livebirth rates were compared between these groups and a comparison group of women without cancer in couples with male factor infertility only.

**Results:**

Compared with retrievals for male factor infertility only (*n* = 81 370), the number of oocytes retrieved was not significantly different for women who underwent retrieval for fertility preservation (*n* = 2941) but was significantly lower for women who underwent retrievals after cancer treatment (*n* = 2479) (mean difference = −2.99, 95% confidence interval [CI] = −3.40 to 2.59). Rate of conception as a function of transfer attempts and likelihood of livebirth after conception also did not significantly differ for fertility preservation (*n* = 291) compared with male factor infertility only (*n* = 34 410). Women with IVF after cancer treatment (*n* = 672) had a lower rate of conception (hazard ratio = 0.70, 95% CI = 0.61 to 0.79) but a similar overall likelihood of a livebirth after conception, relative to the group with male factor infertility only.

**Conclusion:**

IVF outcomes may be maximized when ovarian retrieval is initiated before cancer treatment.

## Introduction

Approximately 60% of reproductive-age (15-44 years) cancer patients want the possibility of future children,^1^ and existing evidence supports the safety of pregnancy after cancer for women^2^^,^^3^ and their offspring.^4^ Yet many cancer treatments, including systemic chemotherapies, cranial or pelvic radiation, and gynecologic surgery, can threaten future fertility.

Since 2006, national guidelines have recommended that reproductive-age patients receive counseling about fertility preservation techniques before administering gonadotoxic therapies.^5-7^ Accepted, nonexperimental options for postpubertal patients are limited to embryo and oocyte cryopreservation, both of which ultimately require in vitro fertilization (IVF) to achieve pregnancy. However, the use of IVF for fertility preservation does not guarantee future ability to have children. In the general population, the percentage of completed egg retrievals that result in a livebirth delivery ranges from about 54% for women younger than 35 to 10% for women older than 40.^8^

Despite the acknowledged need for fertility preservation to address potential late effects of cancer treatment, there is inadequate evidence to allow patients and their providers to make informed decisions. The timing of procedures is critical because IVF procedures initiated before gonadotoxic therapies may have more favorable outcomes than those initiated after. Yet even when oocytes are retrieved before cancer treatment, there is still concern that pathologic changes due to cancer may adversely affect IVF success.

To provide high-quality evidence on clinical parameters surrounding IVF in the cancer context, we used detailed data from a national IVF database linked to statewide cancer registries in 9 states. We investigated oocyte retrieval outcomes, conceptions, and live births among women with cancer compared with women in couples with male factor infertility only as their indication for IVF.

## Methods

### Data sources

Data used for these analyses came from the national Society for Assisted Reproductive Technology Clinic Outcomes Reporting System (SART CORS) database (2004-2018) linked to statewide cancer registries and birth certificate data in 9 states (CA, CO, MA, MD, MI, NC, NJ, NY, VA). SART CORS includes information on demographic factors, IVF indications and treatment parameters, and clinical pregnancy outcomes for IVF procedures from >85% of US clinics.^9^ State cancer registry data were used to define cancer type, age at diagnosis, and first course of treatment (surgery, radiation, chemotherapy). Codes used to define cancer type are provided in Table S1.

This research was approved by the institutional review board at the University of North Carolina and by the CA Center for Health Statistics and Informatics, the CO Central Cancer Registry, the MA Cancer Registry, the MD Cancer Registry, the MI Cancer Registry, the NJ State Cancer Registry and Cancer Surveillance Research Program, the NC State Center for Health Statistics, the NC Advisory Committee on Cancer Coordination and Control, the NY State Cancer Registry and Cancer Statistics, the VA Cancer Registry, and the SART Research Committee.

### Exposure

The primary exposure was the indication for IVF. Using the linked data, we identified all women who had a cancer diagnosis date that preceded the date of first oocyte retrieval. Indication for women with cancer was defined as either fertility preservation or IVF after cancer treatment based on procedure dates (SART does not have standardized fields for cancer-related indications). We defined fertility preservation as oocyte retrieval that was initiated within ≤90 days of a cancer diagnosis. This threshold was defined using the 80th percentile of the distribution of days from cancer diagnosis to first potentially gonadotoxic treatment (any chemotherapy, gynecologic cancer surgery, or radiation for hematologic malignancies or gynecologic cancers). A total of 7501 women with gonadotoxic treatment were identified in the cancer registry data prior to SART linkage. Of these 7017 (95%) had available cancer diagnosis and treatment date information and were used to define the 90-day threshold. Oocyte retrievals initiated >90 days after diagnosis were considered to be IVF after cancer treatment. Among women who did not have a cancer diagnosis, we identified a comparison group of women in couples with male factor infertility only as their indication for IVF.

### Outcomes

Number of oocytes retrieved was used as a continuous value. Conception was defined as a recorded clinical intrauterine gestation in the SART CORS data for the specified IVF cycle. Delivery dates from state birth certificate data were used to identify births resulting from IVF. Those that had a delivery date within 12 months of the start of any IVF SART cycle that had an associated embryo transfer were defined as a birth resulting from the IVF cycle. Live birth was defined based on birth certificate data and was limited to ≥22 weeks gestation and ≥300 grams birthweight.

### Population for analysis

Analyses of the number of oocytes retrieved were limited to a woman’s first autologous retrieval cycle. Of 1 208 304 total cycles, we excluded nonautologous cycles (*N* = 127 597), maternal ages <20 or >49 (*n *= 1536), cycles for women with no retrieval (*n* = 37 499), and cycles that preceded a first retrieval cycle (eg, canceled cycles) *(n* = 32 258). Of the remaining 1 009 414 cycles, 411 860 represented first autologous oocyte retrieval cycles by individual women. Among these, we identified 5420 women with a cancer diagnosis before retrieval and a comparison group of 81 370 women in couples with male factor infertility (Figure S1).

To investigate conception rates for cryopreserved embryos, women with ≥1 thaw-to-transfer cycle using cryopreserved autologous embryos (embryos from autologous oocytes and partner sperm) (*N* = 155 178) were selected. Women were excluded if birth timing in relation to cancer diagnosis and IVF cycle could not be determined due to incomplete date information (*n* = 3). We further excluded those with missing numbers of embryos transferred (*n* = 31) or missing date or indication for the freeze cycle (*n* = 3019) leaving records from a total of 152 125 women available for analysis. Of these, 2802 used a gestational carrier and are described separately. Among the remaining 149 323, analyses included a total of 963 women with a cancer diagnosis before retrieval and a comparison group of 34 410 women in couples with male factor infertility only (Figure S2). Analyses of live birth were conducted among women with a conception. Eligible women with a thaw-to-transfer cycle with tubal ligation as their indication for IVF (*n *= 1504) were retained for sensitivity analyses of conception and live birth.

### Statistical analysis

The number of oocytes retrieved was square root transformed to normalize the distribution, and adjusted mean differences (AMDs) were estimated using multivariable linear regression. First-order Taylor expansion was used to obtain the difference in the estimated square root of the number of oocytes retrieved. The delta method was used to derive asymptotic variance to calculate standard errors and variances.^10^

Multivariable models for the number of oocytes retrieved included adjustment for state, race (Hispanic, non-Hispanic Asian, non-Hispanic Black, non-Hispanic White), age (continuous), and calendar year of retrieval (continuous). Follicle-stimulating hormone (FSH) dosage was missing for approximately 5% of women. In sensitivity analyses, we performed additional adjustment for FSH dosage (continuous) to evaluate whether inclusion mitigated associations with the indication. Anti-Mullerian hormone (AMH; a marker of ovarian reserve) values were routinely recorded by SART after 2012. Therefore, we performed sensitivity analyses adjusting for AMH in the subset of data collected post-2012. Body mass index (BMI) was also frequently missing (34.6%). We conducted sensitivity analyses adjusting for BMI among those with available information and plausible BMI values (15-45 kg/m^2^) (N = 269 294 records).

We calculated the conception rate as a function of the number of thaw-to-transfer attempts. Kaplan-Meier estimators were used to compare conception rates per cycle by exposure category. This conception outcome only considers the first conception and the number of transfer attempts until a first conception.

Multivariable discrete Cox regression models (with transfer cycle number as the time scale) were used to estimate hazard ratios (HRs) for conception. All models were adjusted for maternal age, race/ethnicity, number of embryos transferred (1, 2, or 3+), and duration of freeze (continuous). We repeated sensitivity analyses for BMI as described above.

Multivariable log linear (binomial) regression models were used to estimate risk ratios (RRs) for live birth among women with a conception, adjusting for patient age at thaw, number of embryos transferred, race, and duration of freeze. Sensitivity analyses were conducted for conceptions and live births using women with tubal ligation as their indication as the reference group.

All statistical tests used a 2-sided α = 0.05 and were performed using SAS software, version 9.4.

## Results

### Number of oocytes retrieved

During 2004-2018, we identified 5420 women with a cancer diagnosis before retrieval. Of these, 2941 (54%) were categorized as fertility preservation and 2479 (46%) as IVF after cancer treatment (Table 1). The noncancer comparison group included 81 370 women in couples with male factor infertility only as their indication.

**Table 1.** Characteristics of women’s first autologous oocyte retrieval cycle according to the indication for retrieval.

	Fertility preservation		After cancer treatment		Male factor infertility only	
**Total women**, N, %	2941	100.0%	2479	100.0%	81 370	100.0%
**Number of oocytes retrieved**						
Crude mean (SD)	16.5	(11.3)	11.8	(9.1)	14.5	(8.5)
0	25	0.90%	36	1.50%	303	0.40%
≥1	2916	99.10%	2443	98.50%	81 067	99.60%
**Cycle outcome**						
Oocyte cryopreservation only	954	32.4%	376	15.2%	555	0.7%
Embryo cryopreservation only	1861	63.3%	1262	50.9%	45 894	56.4%
Oocyte and embryo cryopreservation	11	0.4%	NR	NR	157	0.2%
No oocytes successfully retrieved	25	0.9%	NR	NR	275	0.3%
Fresh transfer (no cryopreservation)			599	24.2%	30 926	38.0%
Oocytes retrieved no further data	90	3.1%	202	8.1%	3563	4.4%
**Age at retrieval**						
Mean (SD)	32	(5.2)	35	(5.5)	34	(4.3)
**Race/ethnicity**						
Asian	207	7.0%	225	9.1%	8247	10.1%
Black	115	3.9%	NR	NR	3391	4.2%
Hispanic	NR	NR	121	4.9%	4546	5.6%
Other	NR	NR	NR	NR	102	0.1%
Unknown/not stated	1285	43.7%	990	39.9%	29 435	36.2%
White	1223	41.6%	1032	41.6%	35 649	43.8%
**State**						
CA	809	27.5%	597	24.1%	19 146	23.5%
CO	82	2.8%	46	1.9%	1725	2.1%
MA	265	9.0%	254	10.2%	8153	10.0%
MD	204	6.9%	167	6.7%	5892	7.2%
MI	162	5.5%	134	5.4%	5364	6.6%
NC	184	6.3%	133	5.4%	4459	5.5%
NJ	369	12.5%	366	14.8%	11 883	14.6%
NY	710	24.1%	618	24.9%	18 367	22.6%
VA	156	5.3%	164	6.6%	6381	7.8%
**AMH Level (ng/mL), median (IQR)**	2.6	(1.4-4.7)	1.7	(0.7-3.4)	2.9	(1.7-4.8)
**FSH dosage (IU), median (IQR)**	3000	(2100-4055)	3375	(2375-4500)	2700	(1900-3750)
**Cancer type**						
Breast	1884	64.1%	773	31.2%		
Thyroid	19	0.6%	478	19.3%		
Melanoma	11	0.4%	241	9.7%		
Hematologic malignancy	444	15.1%	273	11.0%		
Gynecologic malignancy	248	8.4%	381	15.4%		
GI/GU	135	4.6%	104	4.2%		
CNS and other intracranial and intraspinal	71	2.4%	64	2.6%		
Osseous and hondromatous	29	1.0%	22	0.9%		
Soft tissue sarcoma	32	1.1%	37	1.5%		
Other	68	2.3%	106	4.3%		
**Age at cancer diagnosis**						
Mean (SD)	32	(5.2)	33	(5.7)		
**Chemotherapy**						
No chemotherapy	426	14.5%	1644	66.3%		
Chemotherapy	2458	83.6%	734	29.6%		
Unknown	57	1.9%	101	4.1%		

Abbreviations: GI = gastrointestinal; GU = genitourinary; NR = not reported to comply with cell size suppression requirements.

Of the 2941 in the fertility preservation group, 2462 (84%) did not return over an average follow-up of 4.2 years (range = 0-14.6). Of those who did not return, 132 (5%) had a natural birth record. The average time from first retrieval to natural birth was 3.4 years (range = 1.1-11.5).

Overall, the mean number of oocytes retrieved was similar for fertility preservation indications compared with male factor infertility only (16.8 vs 16.9 oocytes; AMD = −0.11; 95% confidence interval [CI] = −0.53 to 0.30) (Table 2). However, in analyses by cancer type, women with hematologic cancers (AMD = −0.81; 95% CI = −1.22 to 0.40) and gynecologic cancers (AMD = −2.59; 95% CI = −2.97–2.20) both had a significantly lower number of oocytes in retrievals for fertility preservation.

**Table 2.** Multivariable-adjusted mean number of oocytes retrieved according to the indication for retrieval, overall and by cancer type.

Indication for retrieval	**Adjusted mean number of oocytes** ^a^	**Adjusted mean difference** ^a^	**95% CI** ^a^
**Overall**			
Male factor infertility only	16.91	Referent	
Fertility preservation	16.81	−0.11	(−0.53 to 0.30)
After cancer treatment	14.06	−2.99	(−3.40 to −2.59)
**Breast**			
Male factor infertility only	16.92	Referent	
Fertility preservation	17.38	0.48	(0.06 to 0.91)
After cancer treatment	14.61	−2.42	(−2.84 to −2.00)
**Hematologic**			
Male factor infertility only	16.92	Referent	
Fertility preservation	16.14	−0.81	(−1.22 to −0.40)
After cancer treatment	10.72	−6.53	(−6.89 to −6.16)
**Gynecologic**			
Male factor infertility only	16.93	Referent	
Fertility preservation	14.67	−2.59	(−2.97 to −2.20)
After cancer treatment	13.49	−3.61	(−4.01 to −3.21)
**Thyroid**			
Male factor infertilty only	16.23	Referent	
After cancer treatment	14.34	−2.71	(−3.13 to −2.30)
**Melanoma**			
Male factor infertility only	16.23	Referent	
After cancer treatment	16.27	−0.69	(−1.12 to −0.25)

a Adjusted for state, race, calendar year, and age.

On average, women who initiated oocyte retrieval after cancer treatment had approximately 3 fewer oocytes retrieved than those with male factor infertility only (AMD = −2.99, 95% CI = −3.40 to 2.59). Differences were greatest for hematologic cancer (AMD = −6.53) and gynecologic cancers (AMD = −3.61). Overall findings for all cancer types combined were similar in sensitivity analyses accounting for AMH, FSH, or BMI (data not shown).

### Conception and live births

Table 3 shows characteristics of included women who had a thaw-to-transfer attempt using cryopreserved embryos and were included in analyses of conception rates and live births. We identified 963 women with a cancer diagnosis before retrieval: 291 (30%) were categorized as fertility preservation and 672 (70%) as IVF after cancer treatment. A total of 34 410 women in couples with male factor infertility only as their indication were included as the comparison group.

**Table 3.** Characteristics of women with at least 1 transfer cycle using cryopreserved embryos.

	Fertility preservation		After cancer treatment		Male factor infertility only	
	*n*	%	*n*	%	*n*	%
**Total women**, *N*, %	291	100%	672	100%	34 410	100%
**No. of embryos transferred**						
1	128	44%	369	55%	17 625	51%
2	123	42%	225	34%	12 781	37%
≥3	41	14%	78	12%	4004	12%
**Age at first thaw**						
Mean (SD)	37	(4.6)	37	(4.3)	34	(4.1)
**Duration of freeze (years)**						
Mean (SD)	3.07	(1.9)	0.89	(1.3)	0.83	(1.2)
Median (range)	2.81	(0.05-10.6)	0.32	(0-7.5)	0.3	(0-13.2)
<1 year	32	11%	510	76%	26 096	76%
1-1.9 years	58	20%	60	9%	2855	8%
2-2.9 years	66	23%	48	7%	3154	9%
3-4.9 years	91	31%	39	6%	1863	5%
5+ years	44	15%	15	2%	442	1%
**Race/ethnicity**						
Asian	23	8%	76	11%	1827	11%
Black	NR	NR	NR	NR	1355	4%
Hispanic	12	4%	29	4%	1623	5%
Other	NR	NR	NR	0%	46	0%
Unknown	107	37%	250	37%	12 395	36%
White	140	48%	294	44%	15 164	44%
**Cancer type**						
Breast	188	65%	170	25%		
Thyroid	NR	NR	210	31%		
Melanoma	NR	NR	79	12%		
Hematologic malignancy	46	16%	41	6%		
Gynecologic malignancy	13	5%	97	14%		
GI/GU	14	5%	29	4%		
CNS and other intracranial and intraspinal	NR	NR	14	2%		
osseous and chondromatous	NR	NR	NR	NR		
Soft tissue sarcoma	NR	NR	NR	NR		
Other	NR	NR	26	4%		
**Age at cancer diagnosis**						
Mean (SD)	34	(4.4)	33	(4.9)		

Abbreviations: GI = gastrointestinal; GU = genitourinary; NR = not reported to comply with cell size suppression requirements.

**Figure 1.**
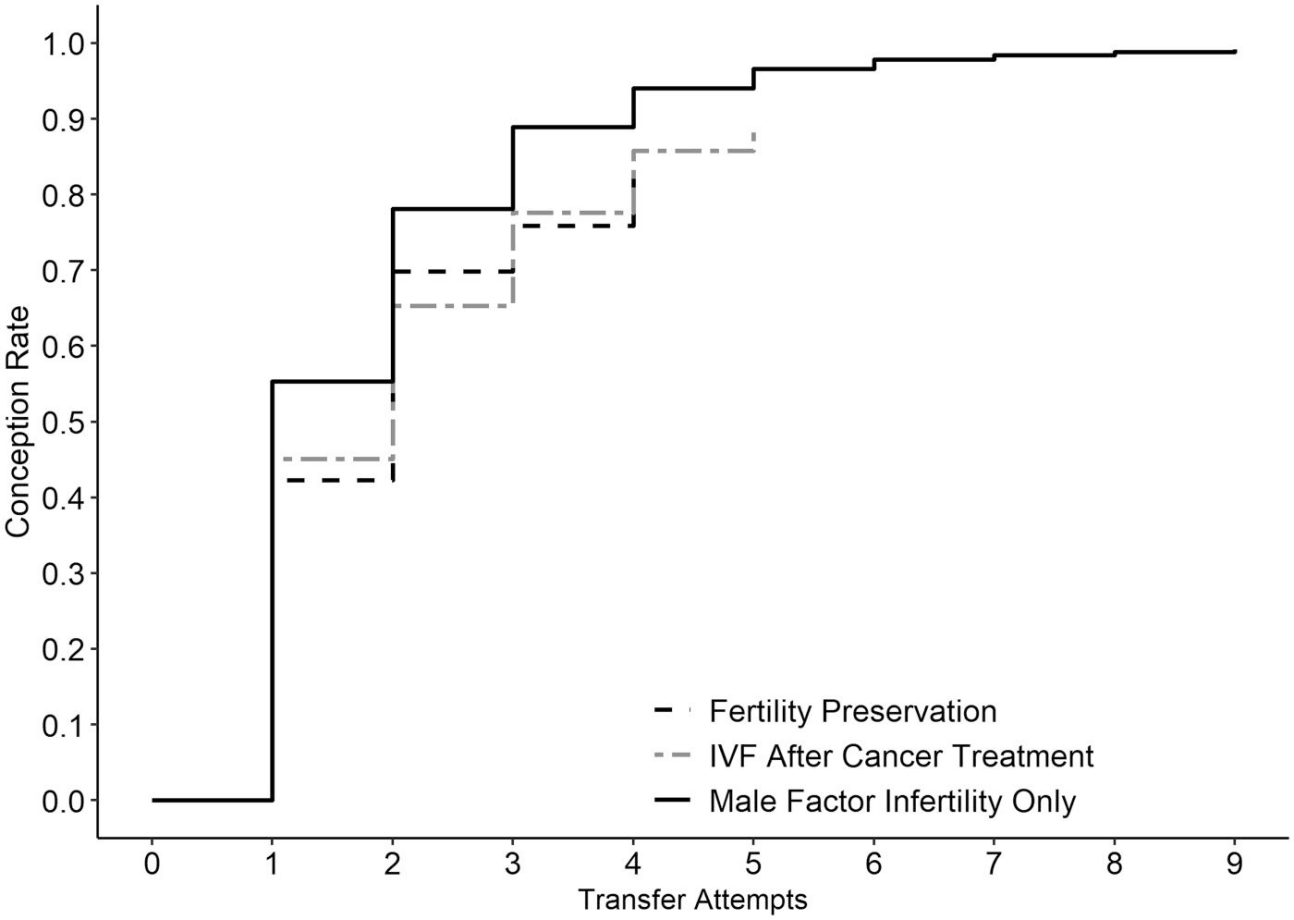
Conception rates as a function of transfer attempts according to in vitro fertilization (IVF) indication.

Cumulative conception rates after 1, 2, and 3 transfer attempts were 42%, 70%, and 76% for fertility preservation, 45%, 65%, and 78% for IVF after cancer treatment, and 55%, 78%, and 89% for male factor infertility only (Figure 1), respectively. In adjusted models of conception rate as a function of transfer attempts, women with fertility preservation before cancer treatment did not significantly differ from women with male factor infertility only at any given level of transfer attempts (HR = 1.16, 95% CI = 0.94 to 1.43) (Table 4). In contrast, those with IVF after cancer treatment had a significantly lower conception rate relative to male factor infertility (HR = 0.70, 95% CI = 0.61 to 0.79). Findings were similar in sensitivity analyses adjusting for BMI (data not shown). In sensitivity analyses with tubal ligation as the reference group (*n* = 1504), the conception rate was significantly higher for women in the fertility preservation group (HR = 1.73, 95% CI = 1.38 to 2.18) but similar for women with IVF after cancer treatment (HR = 1.05, 95% CI = 0.90 to 1.22).

**Table 4.** Multivariable-adjusted hazard ratios (HRs) for conception and risk ratios (RRs) for live birth, overall and stratified according to cancer type.

		Conceptions		Live births	
		Among all women with a transfer attempt		Among those with a conception	
Indication for retrieval	No. of individuals	Conception, *n*	HR (95% CI)^a^	Births, *n*	RR (95% CI)^a^
Male factor infertility only	34 410	24 795	1	13 309	1
**Overall**					
Fertility preservation	291	161	1.16 (0.94 to 1.43)	87	1.13 (0.98 to 1.30)
After cancer treatment	672	426	0.70 (0.61 to 0.79)	228	1.03 (0.95 to 1.13)
**Fertility preservation by cancer type** (compared with male factor infertility only)					
Breast	188	104	1.24 (0.96 to 1.61)	49	0.98 (0.80 to 1.20)
Hematologic	46	24	0.99 (0.58 to 1.70)	14	1.24 (0.90 to 1.71)
Gynecologic	13	NR	0.22 (0.06 to 0.78)	NR	NC
**After cancer treatment by cancer type** (compared with male factor infertility only)					
Breast	170	92	0.64 (0.49 to 0.83)	51	1.13 (0.94 to 1.35)
Hematologic	41	23	0.74 (0.42 to 1.28)	13	1.09 (0.77 to 1.55)
Gynecologic	97	56	0.54 (0.39 to 0.76)	28	0.92 (0.71 to 1.19)
Thyroid	210	140	0.70 (0.56 to 0.88)	73	0.99 (0.85 to 1.16)
Melanoma	79	59	0.91 (0.63 to 1.30)	33	1.08 (0.87 to 1.35)

Abbreviations: CI = confidence interval; NC = not calculated because of small sample size; NR = not reported to comply with cell size suppression requirements.

a Adjusted for patient age at thaw, number of embryos transferred, race, and duration of freeze.

Among women with a conception, the likelihood of a live birth did not significantly differ for fertility preservation (RR = 1.13, 95% CI = 0.98 to 1.30) or IVF after cancer treatment (RR = 1.03, 95% CI = 0.95 to 1.13) relative to male factor infertility (Table 4). Results were similar in analyses stratified by cancer type. In sensitivity analyses, the likelihood of a live birth was similar for both fertility preservation and IVF after cancer treatment compared with tubal ligation (Table S2).

In analyses among women with cancer only, the conception rate was lower for IVF after cancer treatment than fertility preservation (HR = 0.70, 95% CI = 0.51 to 0.94) (Table 5). Among women with a conception, the likelihood of a live birth did not significantly differ between fertility preservation and IVF after cancer.

**Table 5.** Multivariable-adjusted hazard ratios (HRs) for conception and risk ratios (RRs) for live birth among women with cancer.

		Conceptions		Live births	
		Among all women with a transfer attempt		Among women with a conception	
Indication for retrieval	No. of individuals	Conceptions, *n*	HR (95% CI)^a^	Births, *n*	RR (95% CI)^a^
**Overall**					
Indication for retrieval					
Fertility preservation	291	161	1	87	1
After cancer treatment	672	426	0.70 (0.51 to 0.94)	228	0.91 (0.77 to 1.08)
**Fertility preservation—Duration of freeze**					
<1 year	32	19	1.04 (0.43 to 2.50)	11	0.99 (0.79 to 1.24)
1-1.9 years	58	35	0.91 (0.46 to 1.82)	19	0.99 (0.81 to 1.20)
2-2.9 years	66	42	1	23	1
3-4.9 years	91	47	0.44 (0.24 to 0.81)	26	1.01 (0.85 to 1.19)
5+ years	44	18	0.42 (0.20 to 0.90)	NR	1.00 (0.79 to 1.27)
**After cancer treatment—Duration of freeze**					
<1 year	510	336	1.52 (0.93 to 2.50)	188	0.98 (0.70 to 1.36)
1-1.9 years	60	34	0.97 (0.52 to 1.81)	16	0.79 (0.50 to 1.26)
2-2.9 years	48	27	1	16	1
3-4.9 years	39	21	1.28 (0.62 to 2.66)	NR	0.58 (0.29 to 1.15)
5+ years	15	NR	0.95 (0.36 to 2.51)	NR	0.20 (0.03 to 1.30)

Abbreviations: CI = confidence interval; NC= not calculated because of small sample size; NR= not reported to comply with cell size suppression requirements.

a Adjusted for patient age at thaw, number of embryos transferred, race, and duration of freeze.

## Discussion

Evidence of the success of IVF procedures is critical for informed decision making before and after cancer treatment. Using data from a national IVF database linked with records from 9 state cancer registries, we investigated oocyte retrieval outcomes and conceptions and live births after IVF among >5000 women with cancer. For first autologous retrieval cycles, we found that, compared with retrievals for women in couples with male factor infertility only, the number of oocytes retrieved was not significantly different for women who underwent retrieval for fertility preservation before cancer treatment but was significantly lower for women who underwent retrievals after cancer treatment. Rate of conception as a function of transfer attempts and likelihood of live birth after conception also did not significantly differ for the fertility preservation group compared with male factor infertility only. Women with IVF after cancer treatment had a lower rate of conception as a function of transfers, but a similar overall likelihood of a live birth after conception, relative to women in couples with male factor infertility only.

Prior reports suggest that the total number of oocytes obtained during retrieval is a key predictor of ultimate IVF success.^11^^,^^12^ A recent meta-analysis reported no significant difference in the number of oocytes retrieved between women with and without cancer (mean difference = 0.73, 95% CI = −0.44 to 1.90), based on 17 studies that focused on IVF before cancer treatment and included age-adjusted estimates.^13^ However, high heterogeneity was noted. Our study is among the largest to date to examine oocyte retrieval outcomes in the fertility preservation context, allowing us to consider retrievals among all cancers combined, as well as within specific subgroups defined by cancer type. Although we did not observe a significant difference in the number of oocytes retrieved overall, comparing fertility preservation to male factor infertility, we did find that fewer oocytes were retrieved for women with hematologic and gynecologic cancers, suggesting that certain malignancies may negatively affect ovarian function, response to stimulation, or access to ovaries, even before gonadotoxic therapy.

Many female cancer survivors may resume regular menstrual cycles after completion of cancer treatment but exhibit decreased ovarian reserve and subfertility in the years that follow,^14^^,^^15^ prompting initiation of IVF. Yet existing studies that have examined outcomes when IVF occurs after cancer treatment, separate from IVF for fertility preservation before treatment, have often been limited by small sample sizes and/or lack of a noncancer comparison group.^16-19^ Overall, among nearly 2500 women with a retrieval after cancer treatment, we found a significantly lower number of oocytes retrieved compared with male factor infertility only. Interestingly, this finding was largely unchanged with adjustment for AMH, a marker of ovarian reserve, among a subset of women with this information available. Other smaller studies have also found that prior exposure to chemotherapy and/or radiation is associated with fewer total oocytes retrieved.^16^^,^^17^ We could not identify specific cancer treatment-related contributors to fewer oocytes, as we lacked information on details of systemic therapy or radiation. However, we did find that the decrease in number of oocytes was particularly apparent among women treated for hematologic cancers, suggesting potential avenues for future research focused on associations with specific therapies.

Even for women who have embryos or oocytes cryopreserved before starting cancer treatment, it is possible that reproductive outcomes after cancer treatment could be affected by treatment-related damage to organs and/or to cardiovascular or pulmonary systems.^20-23^ According to a recent meta-analysis, women with cancer had a significantly lower likelihood of a clinical pregnancy (*n* studies = 2; odds ratio (OR) = 0.63, 95% CI = 0.41 to 0.98) and live birth (*n* studies = 3; OR = 0.58, 95% CI = 0.36 to 0.94) after embryo transfer compared with women without cancer, in studies that were age-adjusted and limited to women who initiated IVF before cancer treatment.^13^ In contrast, we found that both the rate of conception at a given number of transfer attempts and the likelihood of a livebirth did not significantly differ between women who initiated IVF before cancer treatment and women with IVF for male factor infertility only. Our findings may thus provide some reassurance to women who opt to pursue IVF for fertility preservation before starting cancer therapies, by suggesting that their outcomes may be comparable to similarly aged women without a cancer diagnosis.

In our analyses of conception rates as a function of transfer attempts, those with IVF after cancer treatment had a lower rate of conception than those with male factor infertility only. Yet, among women who had a conception, their overall likelihood of having a recorded live birth was similar. Taken together, these results suggest that, at a given number of embryo transfer attempts, women who initiate IVF after cancer treatment may be less likely to conceive compared with their noncancer counterparts but are not more likely to experience a pregnancy loss if they do conceive. Given the high costs of IVF procedures, often incurred out-of-pocket, the potential for a lower conception rate is a meaningful finding that may inform the counseling of survivors considering IVF in the years after completing therapy. Continued investigation will be needed to understand how exposure to specific cancer treatments may influence these associations.

There are some limitations to our analyses. Detailed cancer treatment information (ie, chemotherapy regimens) was not available. This may be a source of potential heterogeneity in post–cancer treatment IVF outcomes. However, we did have detailed data on IVF procedures and basic cancer characteristics, such as cancer type and age at diagnosis, the information most likely to be readily available to reproductive endocrinology and infertility providers. Cancer treatment dates were not available for all women included in our analyses. We therefore distinguished between fertility preservation and IVF after cancer using a 90-day threshold defined based on the distribution of days from diagnosis to treatment among those who had potentially gonadotoxic treatment and available treatment dates. It is possible that this resulted in some misclassification of indication for IVF among women with cancer. Our data sources also would not capture births that occurred out of state (ie, where state of residence at cancer diagnosis and state of birth do not match).

For young women diagnosed with cancer, decisions about IVF are often time-sensitive and made during a vulnerable period of confronting a cancer diagnosis and treatment. Our findings highlight the importance of timely information to support decisions around fertility preservation before starting therapy, as we observed that outcomes were similar between women who initiated IVF before cancer treatment and women without cancer. They also suggest that women who are considering IVF after cancer treatment should be counseled about potentially lower rates of conception as a function of transfer attempts.

## Supplementary Material

pkaf057_Supplementary_Data

## Data Availability

The data underlying this article were provided under agreements with SART CORS and state health departments. The data cannot be shared publicly due to privacy concerns.
