# Supplementary material for: Fertility preservation and in vitro fertilization (IVF) success rates after cancer
Source: JNCI Cancer Spectr. 2025 Jun 10;9(4):pkaf057. doi: 10.1093/jncics/pkaf057 (PMC12249167; doi:10.1093/jncics/pkaf057)
Supplement: pkaf057_Supplementary_Data [file pkaf057_supplementary_data.docx]

Supplementary Material

| Supplementary Table 1.  Cancer type definitions | | | | |
| --- | --- | --- | --- | --- |
| Cancer categories | Subcategories | Behavior Code | Primary Site | Codes |
| Breast | Breast | 2,3 | C500-C509 | Excluding histologies 9590-9989, 9050-9055, 9140+ |
| Thyroid | Thyroid | 3 | C739 | Excluding histologies 9590-9989, 9050-9055, 9140+ |
| Melanoma | Melanoma | 3 | C000-C809 | Include histologies 8720-8790 |
| Hematologic Malignancy | Lymphomas | 3 | C000-C809 | Include histologies 9591, 9670, 9671, 9673, 9675, 9678-9680, 9684, 9689-9691, 9695, 9698-9702, 9705, 9708, 9709, 9714, 9716-9719, 9727-9729, 9765, 9767 |
|  |  | 3 | C000-C809 | Include histologies 9650-9655, 9659, 9661-9665, 9667 |
|  | Leukemias | 3 | C000-C809 | 9826, 9835-9836 |
|  |  | 3 | C420-C421, C424 | 9811-9818, 9837 |
|  |  | 3 | C000-C809 | 9840, 9861, 9865-9867, 9869, 9871-9874, 9891, 9895-9898, 9910-9911, 9920 |
|  |  | 3 | C000-C809 | 9863, 9875-9876, 9945-9946 |
|  |  | 3 | C000-C809 | 9742, 9800-9801, 9805-9809, 9820, 9831-9834, 9860, 9870, 9930-9931, 9940, 9948, 9963-9964 |
|  |  | 3 | C420-C421, C424 | 9823, 9827 |
|  | Myelomas, etc. | 3 | C000-C809 | 9724, 9731-9734, 9740-9741, 9743-9764, 9766, 9769, 9960, 9965-9967, 9970-9971 |
| Gynecologic Malignancy | Cervical | 3 | C530-C539 | Exclude 9050-9055, 9140, 9590-9992 |
|  | Uterine | 3 | C540-C549 | Exclude 9050-9055, 9140, 9590-9992 |
|  |  | 3 | C559 | Exclude 9050-9055, 9140, 9590-9992 |
|  | Ovarian | 3 | C569 | Include 9060-9065, 9070-9073, 9080-9085, 9090-9091, 9100-9102, 9105 |
|  |  | 3 | C569 | Include 8010-8589 |
|  |  | 3 | C000-C809 | Include 8590-8593 |
| GI/GU | Genitourinary | 3 | C649 | 8010-8589 |
|  |  | 3 | C670-C679 | 8010-8589 |
|  |  | 3 | C620-C629 | 8010-8589 |
|  |  | 3 | C000-C809 | 8590-8593 |
|  |  | 3 | C510-C529, C570-C579, C600-C619, C630-C639, C659, C669, C680-C689 | 8010-8589 |
|  | Gastrointestinal | 3 | C180-C218 | 8010-8589 |
|  |  | 3 | C160-C169 | 8010-8589 |
|  |  | 3 | C220-C221 | 8010-8589 |
|  |  | 3 | C250-C259 | 8010-8589 |
|  |  | 3 | C150-C159, C170-C179, C230-C249, C260-C269 | 8010-8589 |
| Other |  | 3 |  | All other codes |

Supplementary Figure 1. Flowchart for oocyte retrieval analyses.


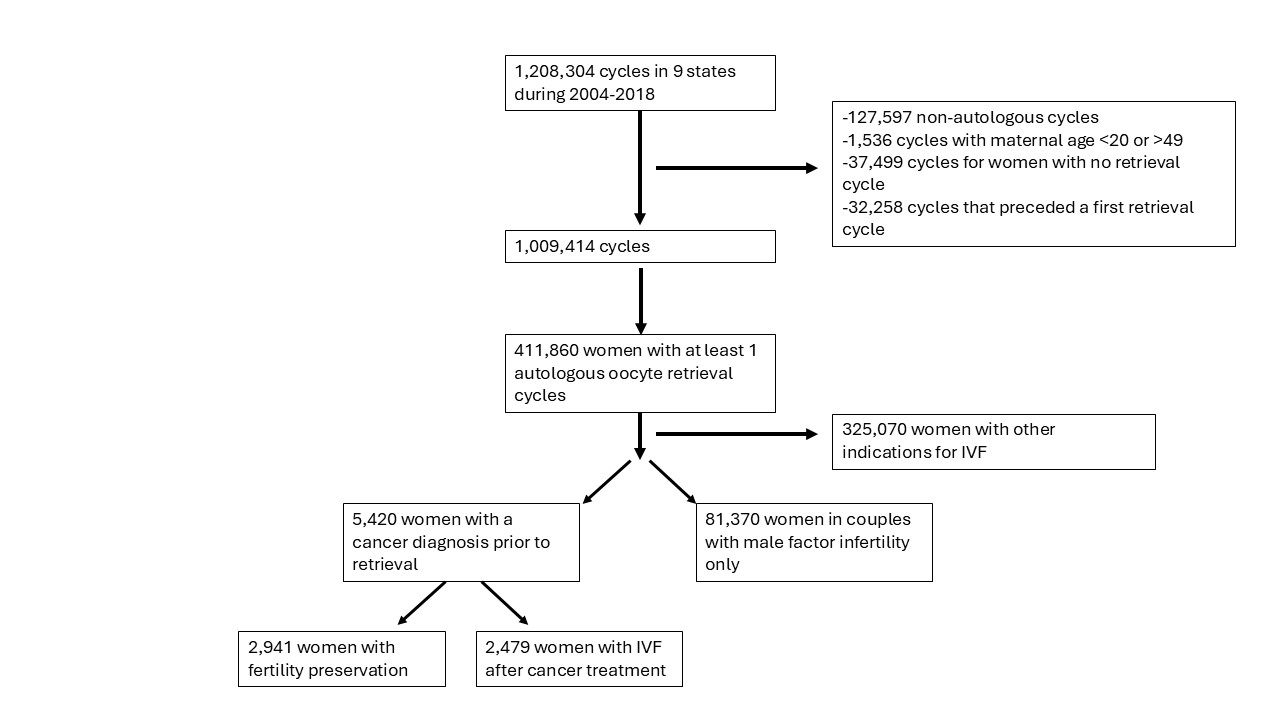


Supplementary Figure 2. Flowchart for conception rate analyses.


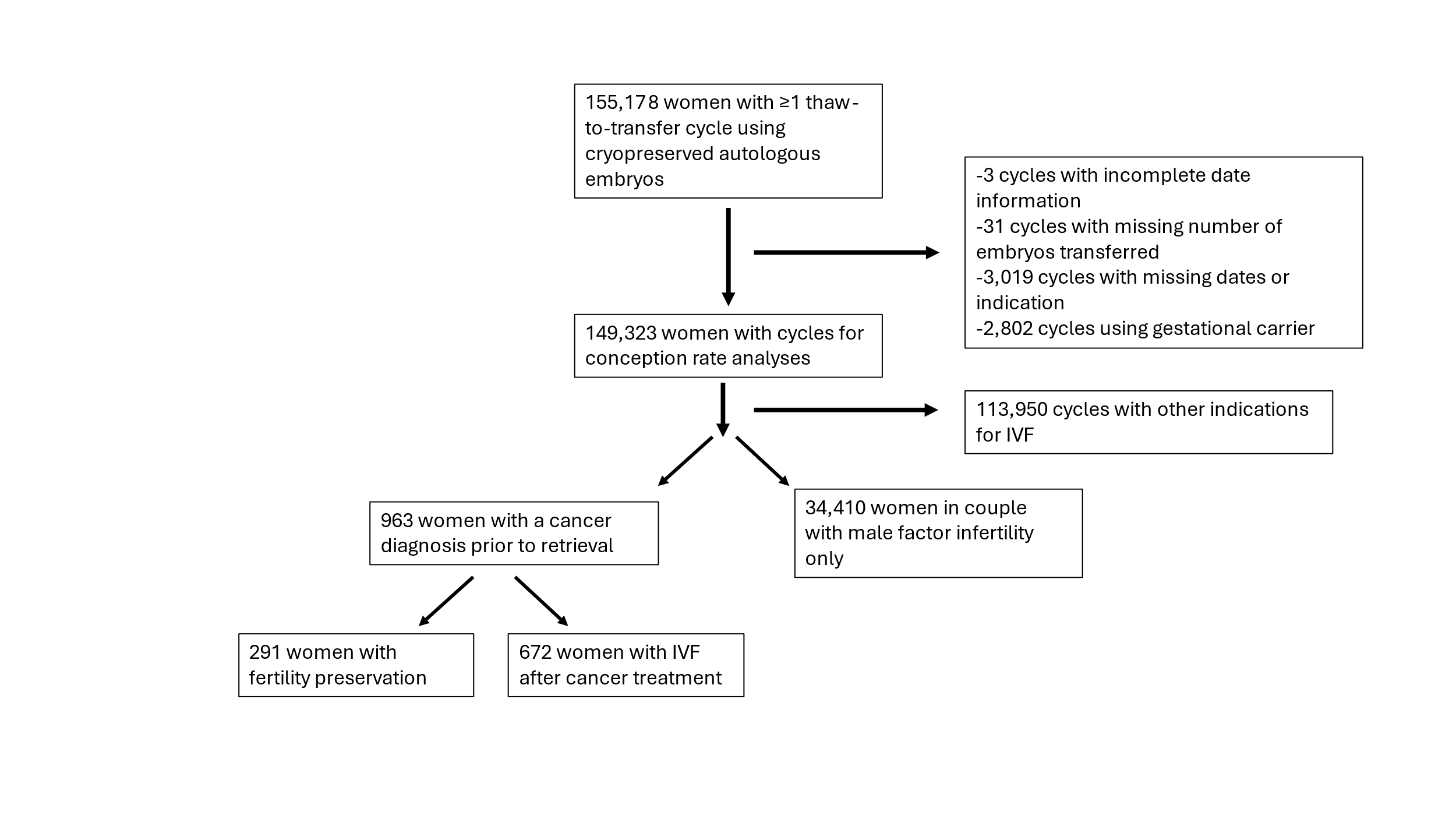


| **Supplementary Table 2.** Multivariable-adjusted hazard ratios (HR) for conception and risk ratio (RR) livebirth, overall and stratified according to cancer type, with tubal ligation as the reference group | | | | | | | |
| --- | --- | --- | --- | --- | --- | --- | --- |
|  |  |  | **Conceptions** | |  | **Livebirths** | |
|  |  |  | Among all women with a transfer attempt | |  | Among those with a conception | |
| Indication for retrieval | N individuals |  | N conceptions | HR (95% CI)^1^ |  | N births | RR (95% CI)^1^ |
| Tubal ligation | 1,504 |  | 828 | 1 |  | 445 | 1 |
|  |  |  |  |  |  |  |  |
| **OVERALL** |  |  |  |  |  |  |  |
| Fertility preservation | 291 |  | 161 | 1.73 (1.38, 2.18) |  | 87 | 1.15 (0.99, 1.35) |
| After cancer treatment | 672 |  | 426 | 1.05 (0.90, 1.22) |  | 228 | 1.05 (0.95, 1.17) |
|  |  |  |  |  |  |  |  |
| **FERTILTY PRESERVATION BY CANCER TYPE** (compared to tubal ligation) | | | | | |  |  |
| Breast | 188 |  | 104 | 1.86 (1.41, 2.45) |  | 49 | 1.00 (0.81, 1.24) |
| Hematologic | 46 |  | 24 | 1.49 (0.86, 2.57) |  | 14 | 1.27 (0.91, 1.76) |
| Gynecologic | 13 |  | NR | 0.33 (0.10, 1.17) |  | NR | NC |
|  |  |  |  |  |  |  |  |
| **AFTER CANCER TREATMENT BY CANCER TYPE** (compared to tubal ligation) | | | | | |  |  |
| Breast | 170 |  | 92 | 0.96 (0.73, 1.27) |  | 51 | 1.15 (0.95, 1.40) |
| Hematologic | 41 |  | 23 | 1.10 (0.63, 1.94) |  | 13 | 1.11 (0.78, 1.59) |
| Gynecologic | 97 |  | 56 | 0.81 (0.57, 1.15) |  | 28 | 0.94 (0.72, 1.22) |
| Thyroid | 210 |  | 140 | 1.36 (0.93, 1.98) |  | 73 | 1.01 (0.85, 1.20) |
| Melanoma | 79 |  | 59 | 1.05 (0.82, 1.34) |  | 33 | 1.10 (0.88, 1.39) |
|  |  |  |  |  |  |  |  |
| Abbreviations: NR= not reported to comply with cell size suppression requirements ; NC= not calculated due to small sample size | | | | | | | |
| ^1^ Adjusted for patient age at thaw, number of embryos transferred, race, and duration of freeze. | | | | | | |  |
